# A genome-wide scan statistic framework for whole-genome sequence data analysis

**DOI:** 10.1038/s41467-019-11023-0

**Published:** 2019-07-09

**Authors:** Zihuai He, Bin Xu, Joseph Buxbaum, Iuliana Ionita-Laza

**Affiliations:** 1 Department of Biostatistics, Columbia University, New York, NY 10032 USA; 2 Department of Neurology and Neurological Sciences, Department of Medicine, Stanford University School of Medicine, Stanford, CA 94305 USA; 3 Department of Psychiatry, Columbia University, New York, NY 10032 USA; 4 Departments of Psychiatry, Neuroscience, and Genetics and Genomic Sciences, Icahn School of Medicine at Mount Sinai, New York, NY 10029 USA

**Keywords:** Next-generation sequencing, Statistics, Autism spectrum disorders

## Abstract

The analysis of whole-genome sequencing studies is challenging due to the large number of noncoding rare variants, our limited understanding of their functional effects, and the lack of natural units for testing. Here we propose a scan statistic framework, WGScan, to simultaneously detect the existence, and estimate the locations of association signals at genome-wide scale. WGScan can analytically estimate the significance threshold for a whole-genome scan; utilize summary statistics for a meta-analysis; incorporate functional annotations for enhanced discoveries in noncoding regions; and enable enrichment analyses using genome-wide summary statistics. Based on the analysis of whole genomes of 1,786 phenotypically discordant sibling pairs from the Simons Simplex Collection study for autism spectrum disorders, we derive genome-wide significance thresholds for whole genome sequencing studies and detect significant enrichments of regions showing associations with autism in promoter regions, functional categories related to autism, and enhancers predicted to regulate expression of autism associated genes.

## Abstract (web summary)

Whole-genome sequencing data reveals a large number of variants for testing their associations with phenotypic traits and diseases. Here, the authors develop WGScan, a statistical method for detecting the existence and estimating the locations of the association signal at genome-wide scale.

## Introduction

Most of the known genetic associations for complex traits have been discovered through genome-wide association studies. With continuing advances in massively parallel sequencing technologies, it becomes increasingly possible to perform large whole-genome sequencing studies, and hence explore the contribution of rare and low-frequency variants in both coding and non-coding regions to risk for complex traits. For example, several large-scale whole-genome sequencing projects are ongoing, including the NHLBI Trans-Omics for Precision Medicine program (TopMed), the NHGRI Genome Sequencing Program (GSP), the UK Biobank and the Simons Simplex Collection^1–4^. The analysis of such data sets is challenging, primarily because of the large number of rare variants in these studies, our inability to accurately predict the functional effects of variants in non-coding regions of the genome, and the lack of natural units (i.e., the analogue of genes in the coding regions) for testing.

Due to the extreme rarity of individual variants, various region-based association tests have been developed to aggregate the genetic variants in a gene/region as opposed to a one-at-a-time single-variant analysis^5^. The analysis of whole-genome sequencing data presents additional challenges due to the lack of natural units for testing in the non-coding part of the genome. Investigators currently adopt a sliding-window strategy to scan the whole genome continuously with a pre-specified window size using region-based tests, and then adjust for multiple testing using Bonferroni correction^6,7^. This strategy is suboptimal because the tests are correlated due to window overlap. Moreover, the optimal window size is often unknown, and misspecification can lead to potential power loss. Alternatively, a less agnostic approach is to test for association with variants in different categories, such as different chromatin states in large number of tissues, gene-based sets etc^8^. A limitation of such analyses is that the choice of categories for testing is subjective, and the correction for multiple testing is limited to the number of categories being tested in individual studies resulting in study-specific significance thresholds.

Here, we propose WGScan, a scan-statistic approach to simultaneously detect the existence and location of the associations in a pre-specified region or at genome-wide scale (more details on existing scan statistics are given in Supplementary Material). We propose an efficient algorithm to estimate moments of the test statistics, such that the significance threshold at genome-wide level can be computed analytically while accounting for the correlation among test statistics. WGScan can incorporate multiple functional annotations of genetic variants for potentially improved power to identify the signals in non-coding regions. Using simulation studies, we show that WGScan outperforms existing methods commonly used in the analyses of whole-genome sequencing data. As a proof of principle, we first apply the proposed WGScan tests to a Metabochip data set on lipid phenotypes focusing on 99 fine-mapping regions (median size 127 kb), and report significant associations between lipid phenotypes and variants in several genes, e.g., *PCSK9, CELSR2, IFT172, LPL, BUD13, CETP, LDLR* and *TOMM40*, with *p*-values substantially smaller than those of standard gene-based tests. We then use data on 7144 whole genomes from the Simons Simplex Collection study on autism spectrum disorders to derive a genome-wide threshold for whole-genome sequencing studies. The method allows a genome-wide scan for both inherited and de novo mutations, complementing previously published papers focusing on de novo variants exclusively^8–10^. We also perform enrichment analyses for the associated regions using several sets, including promoter regions, catalogued gene sets for various complex diseases and traits, enhancers predicted to regulate expression levels for genes in these sets and Gene Ontology (GO) cellular components.

## Results

### Overview of WGScan

We propose a scan-statistic approach, WGScan, for the analysis of whole-genome sequencing data. WGScan can simultaneously detect the existence, and estimate the locations of the association signals at genome-wide scale (or in a pre-specified large region). Specifically, given a study population of *n* subjects, with *Y*_*i*_ being the quantitative/dichotomous outcome value; **X**_**i**_ = (*X*_*i*1_, …, *X*_*id*_)^*T*^ being the *d* covariates, which can include age, gender, principal components of genetic variation etc.; {*G*_*ij*_}_1≤*j*≤*p*_ being the *p* genetic variants in the region of interest, we are interested in simultaneous determining the windows Φ_*kl*_ = {*j*:*k* ≤ *j* ≤ *l*} where the signals reside and testing the association between *Y*_*i*_ and ${\bf{G}}_{{\bf{i}}{\mathbf{\Phi }}_{{\bf{kl}}}}$, adjusting for covariates *X*_*i*_. WGScan is based on score statistics $S_j = \mathop {\sum}\nolimits_{i = 1}^n {G_{ij}\left( {Y_i - \hat \mu _i} \right)}$, *j* = 1, …, *p*, where $\hat \mu _i$ is the estimated mean under the null model.

**Fig. 1:**
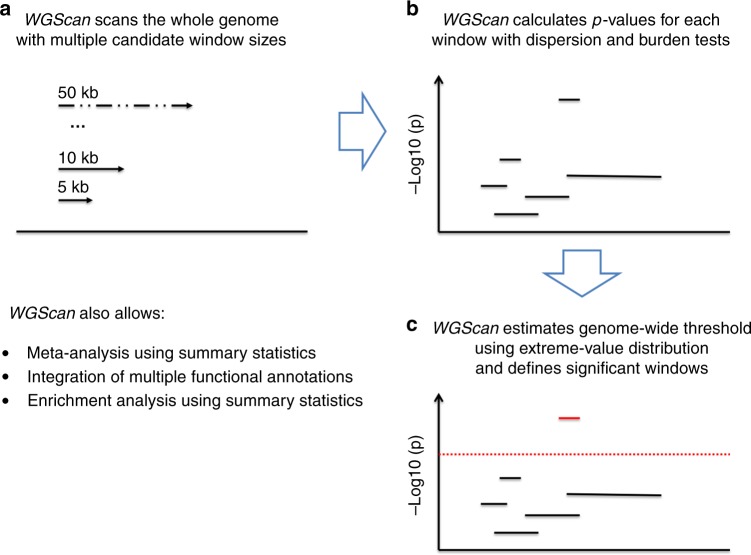
Overview of WGScan

WGScan first scans the genome with two types of scan statistics, dispersion and burden, defined as

1 $$Q_{{\mathrm{Dispersion}},\Phi _{kl}} = \mathop {\sum}\nolimits_{j = k}^l {S_j^2} \,{\mathrm{and}}\,Q_{{\mathrm{Burden}},\Phi _{kl}} = \left( {\mathop {\sum}\nolimits_{j = k}^l {S_j} } \right)^2,$$

and calculates the *p*-values $p_{\Phi _{kl}}$ of both for every window^11–15^. Then it estimates a significance threshold for the minimum *p*-value $\min _{k,l}\left\{ {p_{\Phi _{kl}}} \right\}$, denoted as α^*^, that controls the family-wise error rate of the entire analysis at nominal level (e.g., 0.05). The threshold α^*^ takes into account the multiple testing issue with correlation structure among the test statistics. Finally, it defines all windows Φ_*kl*_ with *p*-values $p_{\Phi _{kl}} < \ \alpha ^ \ast$ as significant. WGScan also allows meta-analysis using summary statistics, integration of multiple functional annotations and enrichment analysis using summary statistics. We present the details in the Methods section. The general workflow of the method is depicted in Fig. 1.

### Empirical family-wise error rate simulations

We first conducted empirical family-wise error rate simulations. Each replicate consists of 2000 individuals with genetic data on 400 genetic variants from a 200 -kb region, simulated using the SKAT package^11^. The SKAT haplotype data set was generated using a coalescent model (COSI), mimicking the linkage disequilibrium structure of European ancestry samples^16^. The simulations focus on variants with MAF < 0.05. We incorporate as weights *w*_*j*_ = *beta*(*MAF*_*j*_, 1, 25) to up-weight rare variants, where *MAF*_*j*_ is the minor allele frequency (MAF) for variant *j*. To investigate whether the proposed tests preserve the desired family-wise error rate, we simulated quantitative/dichotomous phenotypes from the following models

- Quantitative trait: *Y*_*i*_ = *X*_*i*1_ + *ε*_*i*_
- Dichotomous trait: logit_*pi*_ = *α*_0_ + *X*_*i*1_

where *X*_*i*1_ ~ *N*(0, 1), *ε*_*i*_ ~ *N*(0, 1), and they are all independent. For the dichotomous trait, *α*_0_ is determined such that the prevalence is 1%. Then equal numbers of cases and controls were generated. We simulated 10^5^ replicates to examine the family-wise error rate, and compared WGScan with alternative methods employed before in the analysis of whole-genome sequencing data, based on an estimation of the effective number of tests, including those based on the Beta distribution (M-Beta) or spectral decomposition (M-Spectral)^8^. More details on these alternative methods are given in the Methods section.

**Fig. 2:**
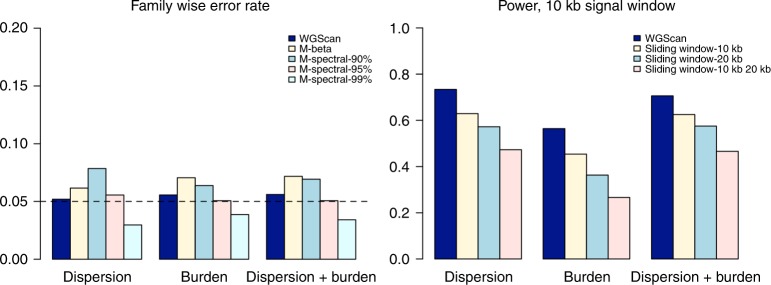
Family-wise error rate and power-simulation studies. The trait is dichotomous, and region size is 200 kb. Several candidate window sizes were considered for WGScan, namely 5 kb, 10 kb, 15 kb, 20 kb, 25 kb and 50 kb. The left panel presents family-wise error rate comparison based on 10^5^ replicates. M-Beta: method based on Beta distribution. M-Spectral-90%/95%/99%: method based on spectral decomposition, where leading eigenvalues account for 90%/95%/99% of the total variation (i.e., sum of all eigenvalues). The right panel presents power comparison based on 1000 replicates, with causal proportion 0.5%. WGScan: proposed test; sliding window: SKAT, Burden or SKAT-O test is applied to scan the region continuously using a sliding window of 10 kb, 20 kb or both, adjusted by Bonferroni correction for the total number of windows tested. Source data are provided as a Source Data file

We present the results for a dichotomous trait in Fig. 2. We observe that the family-wise error rate of WGScan is protected at nominal level. M-Beta exhibits inflated error rate (e.g., 0.0717, combined test), likely due to the violation of the assumption that the correlation among *p*-values only affects the second shape parameter of the beta distribution, which is not theoretically guaranteed. M-Spectral has protected error rate when a correct threshold is specified for the total variation explained (M-Spectral-95%), but it can be either liberal or conservative when a misspecified threshold is being used (e.g., M-Spectral-90% or 99%). The results for quantitative traits are similar (Supplementary Fig. 1).

### Empirical power simulations

We also evaluate the empirical power of WGScan. For each replicate, we generate a 200 -kb region as simulated in the empirical family-wise error rate simulations. We consider two scenarios: high causal proportion with small effects, and low causal proportion with large effects. We set 2.5% or 0.5% variants in the 200 -kb region to be causal, all within a 10 -kb signal window in each scenario. Then we generated the quantitative/dichotomous phenotypes as follows:

- Quantitative trait: *Y*_*i*_ = *X*_*i*1_ + *β*_1_*g*_1_ + … + *β*_*s*_*g*_*s*_ + *ε*_*i*_
- Dichotomous trait: logit_*pi*_ = *α*_0_ + *X*_*i*1_ + *β*_1_*g*_1_ + … + *β*_*s*_*g*_*s*_

where *X*_*i*1_ ~ *N*(0, 1), *ε*_*i*_ ~ *N*(0, 1) and they are all independent; (*g*_1_, …, *g*_*s*_) are selected risk variants. For the dichotomous trait, *α*_0_ is determined such that the prevalence is 1%. Then equal numbers of cases and controls were generated. The sequencing data were generated as described above. We set the effect $\beta _j = a| {\log _{10}m_j} |$, where *m*_*j*_ is the MAF for the *j*th variant. For dichotomous trait, we set *a* = 0.5 when causal proportion is 2.5% (OR = 1.5, when MAF = 0.001), and *a* = 1.2 when causal proportion is 0.5% (OR = 3.6, when MAF = 0.001). For quantitative trait, we set *a* = 0.3 when causal proportion is 2.5%, and *a* = 1.8 when causal proportion is 0.5%.

We compare with the standard sliding-window dispersion (SKAT), burden and combined tests (SKAT-O), using Bonferroni correction^15^. Specifically, we applied dispersion, burden and combined tests to either 10- or 20 -kb sliding windows (sliding window-10 kb/20 kb), with half of the window overlapping with adjacent windows on each side. We also evaluated a sliding-window approach using both 10- and 20 -kb windows (sliding window-10kb20kb), since the underlying signal window is usually unknown. The minimum *p*-value was then adjusted by multiplying by the total number of windows. For all methods, we incorporate MAF-based weights, i.e., *w*_*j*_ = *beta*(*MAF*_*j*_, 1, 25), where *MAF*_*j*_ is the minor allele frequency for variant *j*.

We present results for empirical power for a dichotomous trait and 0.5% causal proportion in Fig. 2. We noticed that sliding window-10 kb exhibits higher power than the sliding window-20 kb in all scenarios, because the underlying signal window is 10 kb, and therefore sliding window-20 kb assumes a misspecified working window size. In addition, sliding window-10 kb/20 kb is less powerful than either sliding window-10 kb or sliding window-20 kb, although the underlying signal window is included. This is because consideration of multiple candidate window sizes substantially increases the burden for multiple comparisons, especially when a Bonferroni correction is applied which does not take into account the correlation and overlap among windows. The proposed WGScan exhibits higher power than all compared methods, including sliding window-10 kb where the working window size is the same as the signal window size. In addition, the power improvement is larger when the causal proportion is lower. There are two reasons for this power improvement. First, WGScan takes into account the correlation and overlap among windows while sliding window-10 kb uses a conservative Bonferroni correction. Second, although the underlying signal window is 10 kb, the 0.5% or 2.5% randomly selected causal variants can be concentrated in smaller windows within the 10 -kb signal window, especially when the causal proportion is low (0.5% in this example). WGScan is able to search for the windows that contain the clustered causal variants. We observed similar qualitative patterns in power comparisons for quantitative traits, as well as the power comparisons with 2.5% causal proportion (Supplementary Fig. 2).

### Meta-analysis of candidate regions in Metabochip data for lipid traits

As a proof of principle, we first applied WGScan to regions potentially associated with total cholesterol (CHOL), high-density lipoproteins cholesterol (HDL), low-density lipoproteins cholesterol (LDL) and triglycerides (TG) using Metabochip data on eight studies collected in Northern and Western Europe, for a total of 12,281 individuals^17^. Details on these studies are reported in Supplementary Material. We restricted the analysis to rare (minor allele frequency < 0.05) variants. We report the results from WGScan, scanning 99 fine-mapping regions (median size 127 kb) with candidate window sizes 5 kb, 10 kb, 15 kb, 20 kb, 25 kb and 50 kb, with half of the window overlapping with adjacent windows on each side. In addition, we performed a gene-based test for 266 genes located in these regions^18^. We aim to illustrate that the proposed scan-statistic approach outperforms the gene-based approach. Therefore, we compared WGScan with gene-based approach in an integrative analysis setting, where both methods include the same set of GenoNet functional predictions across 127 tissues to up-weight predicted functional variants for a fair comparison.^19^

**Fig. 3:**
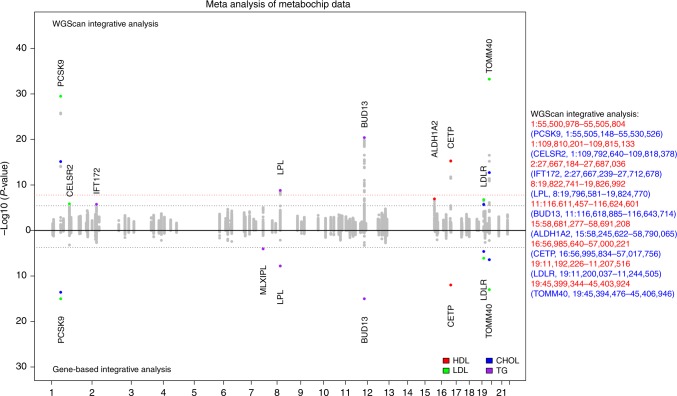
Application to the Metabochip data. The top panel shows the results of WGScan (sliding-window analysis) with candidate window sizes 5 kb, 10 kb, 15 kb, 20 kb, 25 kb and 50 kb. Each dot corresponds to a window. The highlighted dots correspond to significant windows and the overlapping genes. The bottom panel shows the results from the gene-based analyses. Each dot corresponds to a gene. The highlighted dots represent significant genes. For each lipid trait, a significance threshold (3.75e−06; black dashed line) is estimated for the minimum *p*-value of original dispersion and burden tests, and all tests are weighted by GenoNet scores across 127 tissues (in total 256 tests per window). The red dashed line presents the Bonferroni threshold (1.66e−08). The gene-based significance threshold (1.88e−04) is calculated by Bonferroni adjustment based on 266 genes located in the 99 fine-mapping regions. The right panel presents the significant windows (and the overlapping genes) identified by WGScan. Source data are provided as a Source Data file

We present the significant windows and genes in Fig. 3, and the exact *p*-values in Supplementary Table 1. For the WGScan analysis, we estimate a significance threshold of 3.75e−06 for all tests (in total 128 × 2 = 256 tests per window). For the gene-based analysis, we used a significance threshold of 1.88e−04, corresponding to the Bonferroni adjustment for 266 genes. Overall, we find that both the scan-statistic and gene-based analyses detect associations with variants in several well-known genes for lipid traits, including *PCSK9, LPL, LDLR* and *CETP*. In addition, the scan-statistic approach also detects associations with variants in several other genes, not detected by the classic gene-based analysis, such as *CELSR2, IFT172* and *ALDH1A2* (note also that using a Bonferroni adjustment for the total number of tests, i.e., 1.66e−08, would not allow detection of these additional genes). Lead SNPs at 95 loci identified in a large GWAS on blood lipid traits have been shown to be eQTLs in human liver for *CELSR2* (rs629301), *IFT172* (rs1260326) and *ALDH1A2* (rs1532085), adding support to the relevance of these genes to lipid traits^20^.

### Applications to whole-genome sequencing data from the Simons Simplex Collection

We then applied WGScan to whole-genome sequencing data from the Simon Simplex Collection (SSC). SSC is a unique repository of 2600 simplex families, collected by the Simons Foundation Autism Research Initiative (SFARI), in order to study genetic variants that contribute to the overall risk of autism spectrum disorders (ASD)^4^. In this analysis, we have used whole-genome data from the Pilot study + Phase 1, Phase 2, Phase 3–1 and Phase 3-2, consisting of 2076, 2368, 1796 and 904 individuals from 519, 592, 449 and 226 quad families, respectively. Each family consists of one ASD case, one unaffected sibling and their parents. Unlike previous studies on this data set that have focused on de novo variants exclusively, we focus on rare variants (minor allele frequency < 0.05), both inherited and de novo^8^. We adopt a simple analysis strategy, and adjust the offspring genotypes by subtracting the conditional expectation (conditional on parental genotypes) from their genotypes, so that the 3572 cases and controls can be considered as conditionally independent samples. For binary outcomes, we show in Supplementary Material that the score test paired with this adjustment is also directly connected to retrospective paired *t* test for discordant sib-pair association, which takes into account the correlation between siblings within a pair. This approach also takes advantage of the family structure to control for population stratification. After all quality control steps detailed in the Method section, the final data set comprised 1786 ASD cases and 1786 sibling controls and 137,732,715 variants, of which 129,820,320 are rare (minor allele frequency or MAF < 0.05). We apply WGScan to all rare (MAF < 0.05) biallelic single-nucleotide variants adjusting for gender, with candidate window sizes 5 kb, 10 kb, 15 kb, 20 kb, 25 kb and 50 kb. The primary analysis used a *beta*(1, 25) weighting scheme to up-weight rare variants, i.e., *w*_*j*0_ = *beta*(*MAF*_*j*_, 1, 25), where *MAF*_*j*_ is the minor allele frequency for variant *j*^17^.

### Significance thresholds for whole-genome analysis

**Fig. 4:**
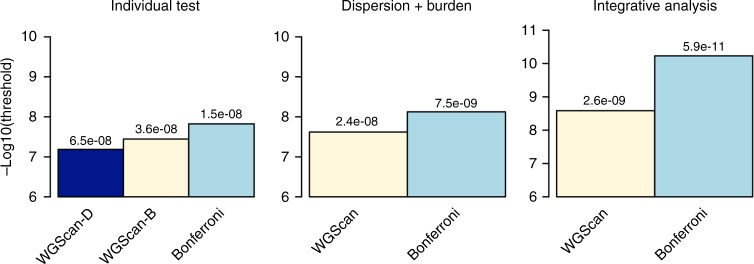
Estimated significance thresholds for whole-genome sequencing studies (based on the Simons Simplex Collection data). Bars present estimated genome-wide significance thresholds (−log10 scale) for different tests. WGScan: WGScan with dispersion and burden tests (two tests per window). WGScan-I: 127 tissue-specific GenoNet scores are integrated, in addition to the original burden and dispersion tests (for a total of 256 tests per window). The significance threshold is estimated for the minimum *p*-value of all tests. The Bonferroni threshold is defined as 0.05 divided by the total number of tests. Source data are provided as a Source Data file

We estimate the significance thresholds for sliding-window approaches with individual dispersion and burden tests, a combination of them (two tests per window), and dispersion and burden tests integrating additional GenoNet scores in 127 tissues (256 tests per window) with candidate window sizes 5 kb, 10 kb, 15 kb, 20 kb, 25 kb and 50 kb, with half of the window overlapping with adjacent windows on each side. The GenoNet functional scores are integrated as additional weights, i.e., *w*_*jr*_ = *beta*(*MAF*_*j*_, 1, 25) × *GenoNet*_*jr*_, *r* = 1, …, 127, where *GenoNet*_*jr*_ is the GenoNet score for variant *j* in *r*th tissue. We present the main results in Fig. 4. As shown, the threshold estimated using WGScan that accounts for correlations among windows (and among functional scores in different tissues/cell types, when they are incorporated) is substantially higher than using a naive Bonferroni threshold, especially when incorporating a large number of functional scores in the analyses (2.6 × 10^−9^ vs. 5.9 × 10^−11^). Interestingly, the threshold when performing only dispersion tests (6.5 × 10^−8^, Supplementary Fig. 3) is slightly higher (more liberal) than when performing only burden tests (3.6 × 10^−8^, Supplementary Fig. 3) due to the fact that generally the correlation among dispersion-type statistics tends to be larger than among burden-type statistics (see also Supplementary Material).

### Scan-statistic analysis of the SSC

We present the genome-wide results of the scan-statistic analysis of 3,090,927 overlapping windows from WGScan in Supplementary Fig. 4. The QQ plot shows that both dispersion and burden test results are concordant with the null expectation in each individual phase. There is no single-window association signal from the standard dispersion or burden tests below the estimated threshold for dispersion and burden tests (2.4 × 10^−8^).

We annotate the location of windows with respect to promoter regions, defined as 3 kb upstream of the transcription start site (TSS) of a gene. In each phase, we computed the proportion of associated windows (at different thresholds *p* < 0.01, 0.005, 0.001 or 0.0005) overlapping promoter regions, and compared it with unassociated windows (*p* > 0.9) using 100,000 resampling replicates. We used Cauchy’s combination test to combine the resulting *p*-values from individual significance thresholds, and then Fisher’s combined probability test to aggregate *p*-values from different phases^21^. The Cauchy’s combination test is applied in order to accommodate the unknown dependency structure among enrichment *p*-values from different significance thresholds. The results are shown in Table 1. We found that the promoter regions are significantly more enriched in associated windows vs. unassociated windows (meta-analysis *p*-value = 5.9e−08 for dispersion test). The significance of enrichment observed in Pilot + Phase 1 data (*p* = 2.00e−05) is replicated in Phase 3–1 (*p* = 4.02e−05) and Phase 3–2 (*p* = 0.0260). Since most variants in the target windows are inherited variants, the effect of variants in promoters in our analysis are mostly attributed to inherited variants, complementing the findings from An et al.^10^ on the effect of de novo mutations in promoter regions.

**Table 1:** Enrichment of association signals in promoter regions

Test	Pilot + Phase 1	Phase 2	Phase 3–1	Phase 3–2	Meta
Dispersion	2.0E−05	1	4.0E−05	0.0260	5.9E−08
Burden	3.2E−05	0.9999	0.9866	0.5377	0.0050

Observed proportion of significant windows that overlap promoter regions are compared with the expected proportion determined empirically based on control windows. The analysis is based on 100,000 replicates

### ToppFun functional enrichment analyses

Using ToppFun, we have also tested whether the association signals from WGScan (windows with dispersion or burden *p* < 0.005 from any individual phase) are enriched in particular Gene Ontology (GO) cellular components, and in sets of genes associated with human diseases^22,23^. We assign a window to a gene if the window overlaps the region 3 kb upstream and downstream of TSS of the gene. The human disease data are based on DisGeNET, a repository of genes and variants associated with human diseases, integrating data from GWAS catalogues, animal studies and expert curated repositories^24^. Currently, DisGeNET contains a large number of associations between 17,074 genes and 20,370 diseases, traits and other clinical phenotypes. Interestingly, we identify significant enrichments in gene sets for substance-related disorders (Burden Bonferroni adjusted *p*: 7.18 × 10^−9^), autistic disorder (4.56 × 10^−8^), autism spectrum disorders 2.59 × 10^−7^) and autosomal recessive predisposition (6.58 × 10^−06^). We also identify significant enrichments among relevant GO cellular components, such as synapse, neuron part, neuron projection, synapse part and cell-projection organisation. Detailed results are shown in Table 2.

**Table 2:** ToppFun enrichment results

	Dispersion			Burden		
Category	Name	*p-*value	Bonferroni *p-*value	Name	*p-*value	Bonferroni *p-*value
Cellular component (GO)	Synapse	6.36E−11	6.00E−08	Synapse	6.52E−23	9.13E−20
	Neuron part	3.12E−09	2.95E−06	Neuron part	4.81E−20	6.73E−17
	Neuron projection	1.27E−08	1.20E−05	Neuron projection	1.75E−19	2.45E−16
	Synapse part	3.19E−08	3.01E−05	Synapse part	1.68E−18	2.36E−15
	Cell projection part	5.47E−08	5.16E−05	Cell projection part	2.21E−17	3.09E−14
Human disease (DisGeNET^a^)	Substance-related disorders	4.50E−13	4.05E−09	Substance-related disorders	4.48E−13	7.18E−09
	Autism spectrum disorders	2.37E−09	2.13E−05	Autistic disorder	2.85E−12	4.56E−08
	Neuroblastoma	4.92E−08	4.42E−04	Schizophrenia	3.95E−12	6.33E−08
	Central neuroblastoma	7.81E−08	7.02E−04	Autism spectrum disorders	1.61E−11	2.59E−07
	Autistic disorder	1.86E−07	1.67E−03	Autosomal recessive predisposition	4.11E−10	6.58E−06

^a^DisGeNET: The DisGeNET database integrates human gene-disease associations from various databases for a large number of Mendelian and complex diseases

### Enrichment of association signals in specific gene/region sets

We then focused on nineteen sets of genes (both ASD associated and general GENCODE-based sets), and an additional set of regulatory non-coding elements to investigate whether the association signals from WGScan are enriched in those sets (more details on these sets are in the Methods section). We also made use of enhancer-target networks from the ENCODE + Roadmap and FANTOM5 data sets for genes in our selected gene sets, resulting in 15 additional sets of enhancer only regions (linked to the genes in the sets of interest) and 15 enhanced gene sets (containing the original genes in the sets and additional enhancer regions predicted to interact with genes in the sets)^25^. We conducted enrichment analyses using window-based summary statistics (*p*-values), as detailed in the Methods section. We present the results in Table 3.

**Table 3:** Enrichment of association signals in 20 target gene sets

	Dispersion				Burden	
Name	GeneSet	EnhancerSet	Enhanced GeneSet	GeneSet	EnhancerSet	Enhanced GeneSet
FMRP_targets_Darnell2011	5.0E−05	0.0016	2.2E−06	0.0057	2.6E−04	6.2E−04
ASD_coexpression_networks_Willsey2013	0.4368	0.8772	0.3939	7.6E−05	0.0013	2.5E−05
Constrained_PLIScoreOver0.9	2.2E-04	0.1319	1.3E−04	1.9E−04	7.0E−04	4.7E−05
FXR2_wt_binding_sites	0.0083	0.0315	0.0012	0.0023	9.9E−05	2.6E−04
FMR1_iso1_wt_binding_sites	0.0254	0.0225	0.0100	0.0133	0.0014	0.0021
FMR1_iso7_I304N_binding_sites	0.0485	0.0088	0.0162	0.0230	4.6E−04	0.0055
FMR1_iso7_wt_binding_sites	0.2504	0.1216	0.1942	0.0226	0.0082	0.0055
Processed_Transcript_GencodeV19	0.0257	—	0.0257	0.0114	—	0.0114
FMR1_iso1_I304N_binding_sites	0.2328	0.0640	0.1521	0.1010	3.3E−04	0.0237
regulatory_elements_neuro	0.0269	—	0.0269	0.3980	—	0.3980
FXR1_wt_binding_sites	0.0611	0.1849	0.0841	0.1087	0.0381	0.1104
BrainExpressed_Kang2011	0.6152	0.0030	0.2729	0.5306	0.0169	0.3241
Protein_Coding_GencodeV19	0.7413	4.4E−04	0.3192	0.4560	0.0363	0.3496
CHD8_targets_Cotney2015_Sugathan2014	0.3743	0.0080	0.3512	0.9345	0.2153	0.9074
Antisense_GencodeV19	0.4770	—	0.4770	0.4183	—	0.4183
lincRNA_GencodeV19	0.9507	—	0.9507	0.4613	—	0.4613
ASD_risk_genes_TADA_FDR0.3	0.7783	0.1752	0.6973	0.4770	0.8620	0.4671
PSD_Genes2Cognition	0.9944	0.0165	0.8704	0.6989	4.6E−04	0.4879
PseudoGencodeV19	1.0000	—	1.0000	0.5864	—	0.5864
Developmental_delay_DDD	0.7380	0.4580	0.6310	0.9987	0.4166	0.9908

For each gene set, we consider three related sets: the original gene set, enhancers predicted to interact with genes in the set, and an enhanced gene set containing both genes and the predicted enhancers. *p*-values from enrichment analyses are reported; those smaller than Bonferroni threshold = 0.05/20 = 0.0025 are bolded

We observed significant enrichment (Bonferroni threshold = 0.05/20 gene sets = 0.0025) via either dispersion test, burden test or both. The top five sets (ranked by minimum *p*-value of dispersion and burden tests on the enhanced gene sets) are: FMRP_targets_Darnell2011 (dispersion: *p* = 5.0e−05); ASD_coexpression_networks_Willsey2013 (burden: *p* = 7.6e−05); Constrained_PLIScoreOver0.9 (burden: *p* = 1.9e−04); FXR2_wt_binding_sites (burden: 0.0023); FMR1_iso1_wt_binding_sites (burden: *p* = 0.0133). The analysis replicated a number of ASD-associated gene sets, concordant with the relevance of these gene sets to autism. Moreover, we identified significant enrichment of associated windows in enhancer regions predicted to interact with these ASD-associated genes (*p* = 0.0016, 0.0013, 7.0e−04, 9.9e−05, 0.0014 for the aforementioned five gene sets). The enhanced gene sets (containing the original genes in the sets and their predicted enhancer regions) generally exhibit smaller *p*-values (*p* = 2.2e−06, 2.5e−05 4.7e−05, 2.6e−04 and 0.0021) than the original gene sets. The results show an effect for enhancers predicted to interact with a number of ASD-associated genes and add support to an important role of non-coding variation in autism.

## Discussion

We propose here a general scan-statistic approach, WGScan, for the analysis of whole-genome sequencing studies. WGScan has several important advantages, namely (1) it scans for association in both coding and non-coding regions using multiple window sizes and multiple functional annotations, while adjusting for the correlation among test statistics, (2) it builds on existing sequence-based association tests such as burden and SKAT, (3) it only needs summary statistics so it can be applied to the meta-analysis of multiple data sets, and (4) it is computationally efficient.

WGScan with one fixed window size can be viewed as an extension of the standard sliding-window approach using SKAT or burden test commonly used in the analysis of whole-genome sequencing data sets, by replacing the standard Bonferroni correction with analytically adjusting for the correlation among test statistics. WGScan improves upon these simple sliding-window analyses by allowing for multiple window sizes, multiple functional annotations, and providing a significance threshold that accounts for the correlations among different windows and annotations.

As illustrated by the two data analyses, WGScan can be flexibly applied to different phenotypes (quantitative and qualitative), different analysis strategies (candidate region analysis and whole-genome analysis) and different study designs (cross-sectional study and family study). Although our analysis focused on rare non-coding variants, we note that the WGScan framework can be readily applied to study common variants or exons solely (by assigning zero weights to rare or noncoding variants respectively). As the quality of variant calling for indels and small structural variants improves, these additional type of variations can be incorporated as well^26^.

Using whole-genome sequencing data from the Simons Simplex Collection study on Autism Spectrum Disorder, we estimate genome-wide significance thresholds that are more liberal than naive Bonferroni thresholds, and therefore can lead to more powerful analyses. This is especially true when integrating a large number of functional annotations; accounting for correlations among different test statistics in this case becomes even more important. For example, the threshold for a whole-genome study using multiple window sizes and multiple functional scores across different tissues/cell types is 2.6 × 10^−9^ using the WGScan approach vs. 5.9 × 10^−11^ when using the naive Bonferroni adjustment.

The application to the Simons Simplex Collection data has led to several interesting results, despite the small sample size of the data set. Namely, analyses based on both rare inherited and de novo variation show autism-associated windows are significantly enriched in several categories, such as promoter regions, gene sets associated with brain-related diseases such as intellectual disability and schizophrenia, and enhancers predicted to interact with these genes. These results support the study of both rare inherited and de novo variation in non-coding regions in autism spectrum disorders using larger sample sizes. The WGScan approach has been implemented in a computationally efficient R package, and can be applied more generally to other whole-genome sequencing studies.

## Methods

### Notations and model

Assume that we have a study population of *n* subjects. Let *Y*_*i*_ be the quantitative/dichotomous outcome value; **X**_**i**_ = (*X*_*i*1_, …, *X*_*id*_)^*T*^ be the *d* covariates which can include age, gender, principal components of genetic variation etc.; {*G*_*ij*_}_1≤*j*≤*p*_ be the *p* genetic variants sequenced in a given region of interest. The model is given by

2 $$g\left( {\mu _i} \right) = {\bf{X}}_{\bf{i}}^{\bf{T}}\alpha + \sum_{j = 1}^p {G_{ij}\beta _j,}$$

where *g* is a link function, *g*(*x*) = *x* for quantitative traits, *g*(*x*) = *logit*(*x*) for binary traits; *μ*_*i*_ = *E*(*Y*_*i*_); *α* and *β* are coefficients for the covariates and genetic variants, respectively. When *β*_*j*_ = 0, there is no association between the trait and *j*th variant. In the proposed approach WGScan, we are interested in simultaneous determining the windows Φ_*kl*_ = {*j*:*k* ≤ *j* ≤ *l*}, where the signals reside and testing the association between *Y*_*i*_ and $G_{i\Phi _{kl}}$, adjusting for covariates **X**_**i**_.

Suppose $S_j = \mathop {\sum}\nolimits_{i = 1}^n {G_{ij}\left( {Y_i - \hat \mu _i} \right)}$ is the score statistic of *j*th variant, where $\hat \mu _i$ is estimated under the null model $g\left( {\mu _i} \right) = {\bf{X}}_{\bf{i}}^{\bf{T}}\alpha$. We consider two types of scan statistics, dispersion and burden, defined as.

3 $$Q_{{\mathrm{Dispersion}},{\it{\Phi }}_{kl}} = \mathop {\sum}\nolimits_{j = k}^l {S_j^2} \,{\mathrm{and}}\,Q_{{\mathrm{Burden}},{\it{\Phi }}_{kl}} = \left( {\mathop {\sum}\nolimits_{j = k}^l {S_j} } \right)^2.$$

It has been shown that asymptotically the dispersion statistic follows a mixture of chi-square distributions, and the burden statistic follows a scaled chi-square distribution^15^. The *p*-value $p_{\Phi _{kl}}$ of both can be calculated as described in the next section. We aim to detect if there is any $p_{\Phi _{kl}}$ below a certain significance threshold that controls family-wise error rate at level *α*. We define an extreme-value statistic as

4 $$T = {\mathrm{max}}_{k,l}\left\{ { - {\mathrm{log}}\,p_{\Phi _{kl}}} \right\}.$$

The (1−*α*)%-quantile of *T* can be estimated by the algorithm described in the next subsection that takes into account the correlations among $\left\{ { - {\mathrm{log}}\,p_{\Phi _{kl}}} \right\}$ due to window overlap, and possible linkage disequilibrium among variants in different windows. Once the (1−*α*)%-quantile, denoted as *T*_1−*α*_, is determined, a significance threshold can be set for minimum *p*-value $\min _{k,l}\left\{ {p_{\Phi _{kl}}} \right\}$, denoted as $\alpha ^ \ast = {\mathrm{exp}}\left( { - T_{1 - \alpha }} \right)$. We define all windows Φ_*kl*_ with *p*-values $p_{\Phi _{kl}} < \ \alpha ^ \ast$ as significant.

It is worth noting that *p*-values are scale-free statistics, therefore the proposed approach based on *p*-values naturally avoids the inflation of scan statistics by purely increasing the window size, an issue discussed in Li et al.^27^. In practice, we search for the optimal window with candidate window sizes 5 kb, 10 kb, 15 kb, 20 kb, 25 kb and 50 kb, with half of the window overlapping with adjacent windows on each side.

Besides identifying individual windows containing variants associated with an outcome of interest, we may be interested in evaluating the overall association between a phenotype and a large candidate region, i.e., if there is any association between the trait and variants in the candidate region. WGScan can also be applied to this setting, and can provide an overall *p*-value for the region, using *p*-value $= \frac{\alpha }{{\alpha ^ \ast }} \times \min _{k,l}\left\{ {p_{\Phi _{kl}}} \right\}$. Here $\frac{\alpha }{{\alpha ^ \ast }}$ can be considered as an estimate of the effective number of tests, and then a Bonferroni-type correction is applied to the minimum *p*-value in the region. This approach is equivalent to the original WGScan approach, because the overall *p*-value $\frac{\alpha }{{\alpha ^ \ast }} \times \min _{k,l}\left\{ {p_{\Phi _{kl}}} \right\} \ < \ \alpha$ is equivalent to at least one window in the region being below the significance threshold, i.e., $\min _{k,l}\left\{ {p_{\Phi _{kl}}} \right\} \ < \ \alpha ^ \ast$.

### Calculation of analytical significance threshold using the Gumbel distribution

Under the null hypothesis, each individual *p*-value $p_{\Phi _{kl}}$ follows a uniform distribution. Therefore $- {\mathrm{log}}\,p_{\Phi _{kl}}$ follows exp (1), an exponential distribution with rate parameter one. We propose to use the Gumbel distribution to approximate the distribution of the extreme-value statistic $T = {\mathrm{max}}_{{\mathrm{k}},{\mathrm{l}}}\left\{ { - \log p_{\Phi _{kl}}} \right\}$. The Gumbel distribution has been used to model the maximum value of random variables following exponential distribution in the statistical theory of extreme values^28^.

5 $$P\left( {T \ > \ x} \right) = 1 - {\mathrm{exp}}\left[ { - {\mathrm{exp}}\left( { - \frac{{x - v}}{\zeta }} \right)} \right],$$

where *E*(*T*) = *v* + *ζγ*; $var\left( T \right) = \frac{{\pi ^2}}{6}\zeta ^2$; *γ* ≈ 0.57721 is the Euler–Mascheroni constant. We can estimate the significance threshold for *p*-values that accounts for the correlation among windows as

6 $$\alpha ^ \ast = {\mathrm{exp}}\left\{ {\zeta {\mathrm{log}}\left[ { - {\mathrm{log}}\left( {1 - \alpha } \right)} \right] - v} \right\},$$

which is the exponential function of the negative (1−*α*)%-quantile of Gumbel distribution (see Supplementary Material for details). We define all windows with *p*-value $p_{\Phi _{kl}} < \ \alpha ^ \ast$as significant. Since the *p*-values $\left\{ {p_{\Phi _{kl}}} \right\}$ are correlated, we estimate *v* and *ζ* by a resampling-based moment-matching approach, described in the next section. Unlike the usual permutation/perturbation-based methods, the proposed method only uses the resampling method to estimate moments of the test statistic so that the *p*-value can still be calculated analytically. We note that a threshold for the combination of dispersion and burden tests while accounting for their correlations can be easily calculated. By defining the extreme-value statistic as

7 $$T = {\mathrm{max}}_{k,l}\left\{ { - {\mathrm{log}}\,p_{{\mathrm{Dispersion}},\Phi _{kl}}, - {\mathrm{log}}\,p_{{\mathrm{Burden}},\Phi _{kl}}} \right\},$$

the proposed inference using Gumbel distribution can be directly applied.

### Resampling-based moment matching approach

We propose the following resampling algorithm to estimate the significance threshold α^*^.

1. Fit the null model and calculate residuals $Y_i - \hat \mu _i$ for *i* = 1, …, *n*.
2. Permute residuals for *B* replicates and construct a *p* × *B* resampling score matrix Δ, with its bth column $\tilde S_b = \mathop {\sum}\nolimits_{i = 1}^n {G_{ij}\left( {Y_{bi} - \hat \mu _{bi}} \right)}$, 1 ≤ *b* ≤ *B*.
3. Construct statistics $\{ {Q_{b,\Phi _{kl}}} \}_{1 \le b \le B}$ for all *k* and *l*; estimate their sample mean $\hat \mu _{\Phi _{kl},B}$, variance $\hat \sigma _{\Phi _{kl},B}^2$ and kurtosis $\hat \kappa _{\Phi _{kl},B} = \hat \psi _{\Phi _{kl},B,4}/( {\hat \sigma _{\Phi _{kl},B}^2} )^2 - \, 3$ where $\hat \psi _{\Phi _{kl},B,4}$ is the sample fourth central moment; then calculate the corresponding *p*-values $\{ {p_{b,\Phi _{kl}}} \}_{1 \le b \le B}$ using moment matching $P_{H_0}( {Q_{\Phi _{kl}} > \ x} ) = 1 - F( {( {x - \hat \mu _{\Phi _{kl},B}} )\sqrt {2df} /\hat \sigma _{\Phi _{kl},B} + df{\mathrm{|}}\chi _{df}^2} )$, where $F( \cdot |\chi _{df}^2)$ is the distribution function of $\chi _{df}^2$ and $df = 12/\hat \kappa _{\Phi _{kl},B}$^15^.
4. Calculate $T_b = {\mathrm{max}}_{k,l} \{ { - {\mathrm{log}}\,p_{b,\Phi _{kl}}} \},1 \le b \le B$; estimate their sample mean, variance and therefore compute $\hat v$ and $\hat \zeta$.
5. Calculate significance threshold $\alpha ^ \ast = {\mathrm{exp}}\left\{ {\hat \zeta \,{\mathrm{log}}\left[ { - {\mathrm{log}}\left( {1 - \alpha } \right)} \right] - \hat v} \right\}$.

It is worth noting that the permutation step only needs to be done once under the null hypothesis where no genetic variants are involved. In addition, the resampling method is only used to estimate the moments of the test statistic, which requires a relatively small value for *B* (in practice we choose *B* = 5000). Therefore, the resampling approach is computationally efficient, as illustrated in a section below regarding computation time. For significant windows, investigators can increase the number of replicates *B* (e.g., 50,000) to reduce the variation in *p*-values due to resampling in step 3. We present the concordance between *p*-values from two replication analyses in Supplementary Fig. 5, with varying number of resampling replicates.

For a genome-wide scan, the estimation of significance threshold (as a function of the genome-wide minimum *p*-value) requires sufficiently accurate calculation of the *p*-values $\left\{ {p_{b,\Phi _{kl}}} \right\}_{1 \le b \le B}$, especially at extreme levels. However, the moment-matching approach in step 3 is an approximation up to the fourth moment. We observe that directly applying the aforementioned approach to the whole genome underestimates the threshold (too liberal), due to the very large number of windows (see Supplementary Fig. 6). In practice, we propose a hybrid Bonferroni-type correction. We divide the genome into 200 -kb regions and apply the WGScan approach to each such region to calculate $\alpha _m^ \ast$. We define $\frac{\alpha }{{\alpha _m^ \ast }}$ as the number of effective tests for each 200 -kb region, and estimate the significance threshold as $\frac{\alpha }{{\mathop {\sum }\nolimits_m \frac{\alpha }{{\alpha _m^ \ast }}}} = \frac{1}{{\mathop {\sum }\nolimits_m \frac{1}{{\alpha _m^ \ast }}}}$. In Supplementary Fig. 6, we show that the threshold estimated by this resampling-based hybrid Bonferroni-type correction is consistent, in the case of the burden test, with unified model-based inference, where we directly apply WGScan to the whole genome but replace step 3 by a model-based inference that is more accurate at extreme *p*-values (e.g., chi-square test for burden test). We only focus on the comparison for burden tests because existing methods to calculate the *p*-value of model-based dispersion test (e.g., Davies’ method) can lead to either inflated or conservative results at extreme levels given the limited sample size of the whole-genome data that we used to estimate the threshold^15^.

### Meta-analysis using summary statistics

The resampling-based moment matching approach only requires summary statistics. Therefore, WGScan can be used for a meta-analysis without sharing of individual level data. The investigators can implement steps 1 and 2 of the resampling algorithm in each study separately to generate original *p*-dimensional score vector *S*^*d*^ and the resampling-based *p* × *B* score matrix Δ^*d*^ from the *d*th study, 1 ≤ *d* ≤ *D*. We construct the overall score vector **S** = **S**^1^ + … + **S**^**D**^ and resampling-based score matrix *Δ* = *Δ*^1^ + … + *Δ*^*D*^. Then steps 3–5 of the resampling-based moment-matching approach can be directly applied. As discussed, the resampling method is only used to estimate the moments of the test statistic, which requires a relatively small *B* (e.g., 5000), resulting in a *p* × *B* matrix of summary statistics.

### Integrative test incorporating multiple functional annotations

We have recently shown how incorporating multiple functional annotations of genetic variants in sequence-based association tests can lead to improved power^25^. The proposed scan-statistic method is able to incorporate multiple functional annotations for improved power. We define

8 $$Q_{{\mathrm{Dispersion}},r,\Phi _{kl}} = \mathop {\sum}\nolimits_{j = k}^l {w_{jr}^2S_j^2} \,{\mathrm{and}}\,Q_{{\mathrm{Burden}},r,\Phi _{kl}} = \left( {\mathop {\sum}\nolimits_{j = k}^l {w_{jr}S_j} } \right)^2$$

where *w*_*jr*_ is the weight based on the *r*th functional annotation for variant *j* (e.g., tissue-specific functional predictions from FUN-LDA (ref) or GenoNet (ref)), which still follows a mixture of chi-square distributions or a scaled chi-square distribution. The extreme-value statistic becomes $T = {\mathrm{max}}_{r,k,l}\left\{ { - {\mathrm{log}}\,p_{r,\Phi _{kl}}} \right\}$. The proposed inference using Gumbel distribution can be directly applied.

### Software implementation and computation time

To facilitate future analyses of whole-genome sequence data, we developed a computationally efficient R package WGScan *(*https://cran.r-project.org/web/packages/WGScan*)*. The package is designed for two common study designs: candidate-region analysis and genome-wide scan. We calculate the average time to analyse a 200 -kb region (~4000 variants on average) generated using the SKAT package with candidate window sizes 5 kb, 10 kb, 15 kb, 20 kb, 25 kb and 50 kb, based on 1000 replicates. We evaluated the utility of the WGScan package, including computational time and peak RAM used in a cluster environment (Intel(R) Xeon(R) CPU E5–2630 v4 @ 2.20 GHz or similar). It mimics the scenario in practice where parallel computing on a cluster is often needed for the analysis of whole-genome sequencing data. We present the results in Supplementary Table 2. The results are for quantitative traits, but those for dichotomous traits are similar.

We observed that both the computational time and peak RAM is increasing as the sample size increases at a rate similar to linear, which makes WGScan scalable to future large-scale whole-genome sequencing studies. When the sample size is 5000, WGScan takes 12.90 s and 1.26 GB (peak RAM) to scan a 200 -kb region using both dispersion and burden tests, with candidate window sizes 5 kb, 10 kb, 15 kb, 20 kb, 25 kb and 50 kb. When the sample size is 100,000, WGScan takes 112.34 s and 14.09 GB (peak RAM) to scan a 200 -kb region.

### Enrichment analyses based on summary statistics

Based on the results from the WGScan approach, it is possible to perform enrichment analyses to test whether association signals are enriched among genes in specific gene sets, or among functional variants in specific tissues or cell types. In this section, we describe a method to enable such enrichment analyses based on the summary statistics (*p*-values for individual windows) generated from WGScan.

Let $Z_{\Phi _{kl}}$ be the inverse-normal transformation of 1−*p*-value corresponding to window Φ_*kl*_ based on a dispersion/burden test from WGScan analysis of a whole-genome sequencing data set. To account for the confounding effect of window size, we first regress $Z_{\Phi _{kl}}$ on the size (in base pairs) of window Φ_*kl*_, and then use the residuals $\tilde Z_{\Phi _{kl}}$ as new outcomes. For a specific gene set, we define $R_{\Phi _{kl}}$ to be the 0/1 indicator of whether window Φ_*kl*_ overlaps with any target gene/region in the set. For a specific tissue or cell type, $R_{\Phi _{kl}}$ is a functional score corresponding to window Φ_*kl*_ (e.g., maximum or mean value of functional scores for the tissue/cell type for the variants in window Φ_*kl*_). We consider the regression model

9 $$E\left( {\tilde Z_{\Phi _{kl}}} \right) = \beta _{r,0} + \beta _rR_{\Phi _{kl}},$$

where *β*_*r*_ quantifies the effect size for the association between window significance and functional score for tissue/cell type *r*. When *β*_*r*_ > 0, windows overlapping the gene set (in gene set analysis), or with larger functional scores (in the tissue/cell type analysis) are more likely to contain association signals. Therefore, we test *H*_0_:*β*_*r*_ = 0 against *H*_1_:*β*_*r*_ > 0 by a Z-test. Due to the correlation among windows, we estimate *β*_*r*_ and the standard deviation by a block jackknife with blocks consisting of 20,000 adjacent windows. That is, we calculate *β*_*r*,*j*_ from *j*-th jackknife replicate where the *j*th 20,000 adjacent windows are removed from the sample, *j* = 1, …, *J*. Then the Z-test statistic is $\frac{{\hat \beta _r}}{{sd\left( {\hat \beta _r} \right)}}$, where $\hat \beta _r = \frac{1}{J}\mathop {\sum}\nolimits_{j = 1}^J {\beta _{r,j}}$, and standard deviation $sd\left( {\hat \beta _r} \right) = \sqrt {\frac{{J - 1}}{J}\mathop {\sum}\nolimits_{j = 1}^J {\left( {\beta _{r,j} - \hat \beta _r} \right)^2} }$. In the analysis of the whole-genome sequencing data from the Simon Simplex Collection (SSC), we applied this enrichment analysis to compare associated windows (determined by *p* < 0.01, 0.005, 0.001 or 0.0005) with unassociated windows (*p* > 0.9). We used Cauchy’s combination test to combine the resulting *p*-values from individual significance thresholds, and then Fisher’s combined probability test to aggregate *p*-values from different phases^21^.

### Alternative methods to calculate the significance threshold

1. *Approach based on the Beta distribution*: For *n* independent *p*-values, *p*_1_, …, *p*_*n*_ ∼ *U*(0, 1), the minimum *p*-value $\min \left( {p_1, \ldots ,p_n} \right)\sim {\mathrm{Beta}}\left( {1,n} \right)$. When *p*_1_, …, *p*_*n*_ are dependent, Werling et al.^8^ assume $\min \left( {p_1, \ldots ,p_n} \right)\sim {\mathrm{Beta}}\left( {1,\hat n} \right)$, and propose to estimate the number of effective tests $\hat n$ using a resampling algorithm, $\hat n = \frac{B}{{m_1 + \ldots + m_B}} - 1$ where *m*_*b*_ is the minimum *p*-value in the *b*th replicate^8^. Then the significance threshold is 0.05/$\hat n$. This adjustment assumes that the correlation among *p*_1_, …, *p*_*n*_ only affects the second shape parameter of the beta distribution, which is not theoretically guaranteed^29^. The violation of this assumption can lead to inflated type I error rate (Fig. 2).
2. *Spectral decomposition*: For *n* *p*-values, *p*_1_, …, *p*_*n*_, we first calculate the corresponding *Z* scores using the inverse-normal transformation. Then we calculate the correlation matrix of the *Z* scores using B resampling replicates as described in the resampling algorithm above, and define $\hat n$ as the number of leading eigenvalues that account for 90–99% of the total variation (i.e., sum of all eigenvalues). Then the significance threshold is *α*/$\hat n$. This adjustment requires specifying a threshold for the total variation explained, but the optimal threshold is often unknown in real data analysis. A misspecified threshold can lead to either inflated or conservative type I error rate (Fig. 2). In addition, using this approach to estimate the number of effective tests $\hat n$ when it is extremely large (e.g., in a genome-wide scan) requires extremely large number of resampling replicates. Therefore, this approach is not computationally scalable for a genome-wide scan.

### The Metabochip data for lipid traits

The Metabochip data set includes data from eight studies consisting of 12,281 individuals: 2793 from HUNT and Tromso in Norwegian; 1529 from DIAGEN in German; 2741 from FUSION stage 2, 2108 from D2D-2007, 429 from DPS, 1439 from METSIM and 1242 from Drs EXTRA in Finnish. DNA samples were genotyped on the Metabochip, a custom genotyping array that includes SNPs to fine map 257 known associations for cardiometabolic traits^30^. Genotyping was performed at the Center for Inherited Disease Research (CIDR) and genotypes called with BeadStudio Genotyping Module, v.3.3.7.

The two Norwegian cohorts were analysed jointly, with a covariate for study origin. We adjusted for gender, age, age squared, type 2 diabetes status for each individual study except for METSIM, which is comprised only of males. We additionally adjusted for birthplace for FUSION stage 2. The covariates adjustment is consistent with Lee et al.^17^. We excluded samples and SNPs with call rates <98% and used the complete data for each trait when there are missing outcomes. We applied normal quantile transformation to each trait and evaluated 266 genes located in the 99 fine-mapping regions (mean size 192,771 bps), meta-analysing the summary statistics from the individual studies. We used *beta*(1, 25) weights to up-weight rare variants, i.e., *w*_*j*_ = *beta*(*MAF*, 1, 25), where MAF is the minor allele frequency for variant *j*. For each trait, a significance threshold is estimated for original dispersion and burden tests combined (in total two tests per window), scanning all regions, with candidate window sizes 5 kb, 10 kb, 15 kb, 20 kb, 25 kb and 50 kb, with half of the window overlapping with adjacent windows on each side.

### Quality control for the whole-genome sequencing data set from SSC

We focus on variant- and genotype-level quality metrics obtained from the VCF^8^. We exclude variants with QUAL (quality score) <200 or ReadPosRankSum <−1.4 (rank-sum test for relative positioning of reference to alternative alleles within reads) or SOR (strand bias estimated by the symmetric odds ratio test) >2.5 or GQ_MEAN (genotype mean quality) <50.0 or QD (quality by depth) <10.0 or AN (allele number) <4152. For heterozygous genotypes, we exclude genotypes (these values will be treated as missing) with GQ (genotype quality of individual samples) <99 or DP (coverage) <10 or AB (allele balance) <0.22 or AB > 0.78. For homozygous genotypes, we exclude genotypes with GQ (genotype quality of individual samples) <30 or DP (coverage) <18 or AB (allele balance) <0.95. The thresholds were selected by Werling et al.^8^ through assessment of sequential receiver-operating characteristic (ROC) curves generated from the true positive and true negative calls for each quality metric^8^.

### Gene sets for the enrichment analyses

We selected 20 sets of genes/regions for our enrichment analyses. The first 13 sets were previously summarised and evaluated by Werling et al.^8^, including: ASD risk genes (FDR < 0.3) obtained from Sanders et al.^31^; genes coexpressed with ASD risk genes identified by Willsey et al.^32^; genes associated with developmental delay from the Development Disorder Genotype-Phenotype Database; CHD8 target genes defined as the union of lists from two ChIP–seq studies; FMRP target genes selected from Darnell et al.^37^; human postsynaptic density (PSD) proteins from the Genes2Cognition database (http://www.genes2cognition.org/); brain expressed genes from Kang et al.^38^; constrained genes defined as having a probability of loss-of-function intolerance (pLI) score ≥ 0.9 in the ExAC database; five categories defined by GENECODE (wgEncodeGencodeCompV19): Protein-coding, Pseudo genes, LincRNA, Antisense and Processed Transcript^8,31–40^. We also selected targeted regulatory noncoding elements from Short et al., including 4307 highly evolutionarily conserved noncoding elements (CNEs), 595 experimentally validated enhancers, and 1237 putative heart enhancers^41^. The remaining six sets correspond to the binding sites within the mRNA targets for wild-type and I304N mutant FMRP isoforms and its paralogs, FXR1 and FXR2^42^.

### Web resources

Annovar: http://annovar.openbioinformatics.org/en/latest/.

Chipseeker: https://guangchuangyu.github.io/software/ChIPseeker/.

Eigen/Eigen-PC: http://www.columbia.edu/~ii2135/eigen.html/.

GenoNet: http://www.funlda.com/genonet/.

SKAT: https://www.hsph.harvard.edu/skat/.

Toppgene: https://toppgene.cchmc.org/.

### Reporting summary

Further information on research design is available in the Nature Research Reporting Summary linked to this article.

## Supplementary information

Supplementary Information

Reporting Summary

Source Data

## Data Availability

All data generated during this study are included in this published article (and its Supplementary Information files). Approved researchers can obtain the SSC population data set described in this study (https://www.sfari.org/resource/simons-simplex-collection/) by applying at https://base.sfari.org. All other relevant data are available from the corresponding author on reasonable request.

## Code availability

We developed a computationally efficient R package WGScan. The package is available at the Comprehensive R Archive Network (CRAN): https://cran.r-project.org/web/packages/WGScan.
