# Supplementary material for: A genome-wide scan statistic framework for whole-genome sequence data analysis
Source: Nat Commun. 2019 Jul 9;10:3018. doi: 10.1038/s41467-019-11023-0 (PMC6616627; doi:10.1038/s41467-019-11023-0)
Supplement: Supplementary file 1 — Supplementary Information [file 41467_2019_11023_MOESM1_ESM.pdf]

A genome-wide scan statistic framework for whole-genome sequence data analysis

He et al.

Supplementary Information for "A genome-wide scan statistic framework for whole-genome sequence data analysis"

Zihuai He[1,2], Bin Xu[3], Joseph Buxbaum[4], Iuliana Ionita-Laza[1, #]

[1]Department of Biostatistics, Columbia University, New York, NY 10032

[2]Department of Neurology and Neurological Sciences, Department of Medicine, Stanford University School of Medicine, Stanford, CA 94305

[3]Department of Psychiatry, Columbia University, New York, NY 10032

[4]Departments of Psychiatry, Neuroscience, and Genetics and Genomic Sciences, Icahn School of Medicine at Mount Sinai, New York, NY 10029

[#]Correspondence to Iuliana Ionita-Laza: ii2135@cumc.columbia.edu

Supplementary Figures

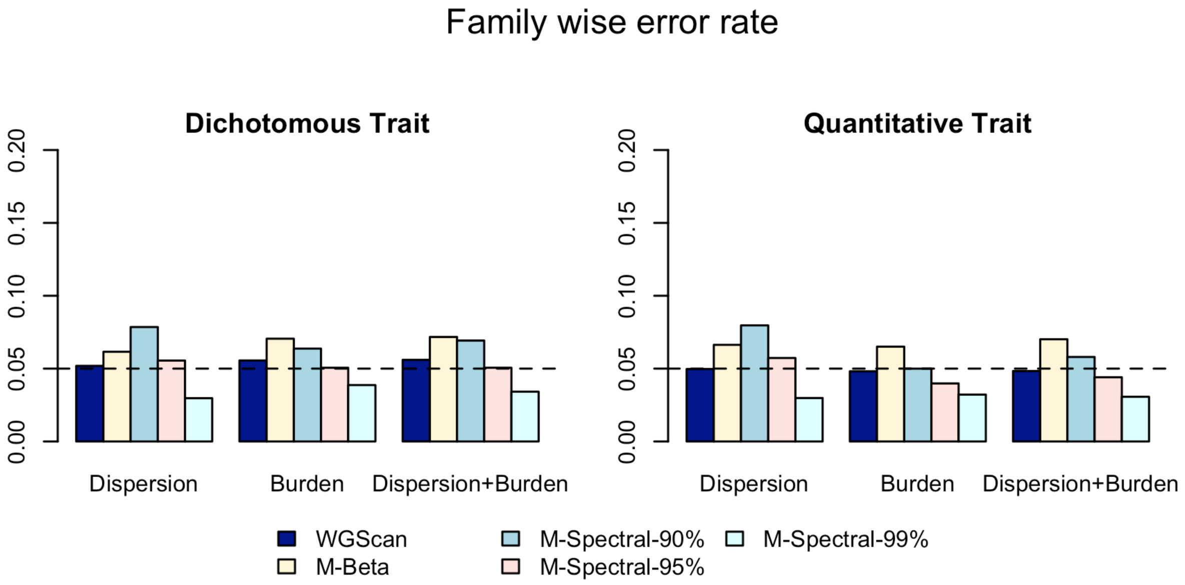

Supplementary Figure 1 Family-wise error rate simulation studies of different methods calculating significance threshold based on $\mathbf{10^5}$ replicates. Several candidate window sizes were considered, namely 5kb, 10kb, 15kb, 20kb, 25kb, 50kb. M-Beta: method based on Beta distribution. M-Spectral-90%/95%/99%: method based on spectral decomposition, where leading eigenvalues account for 90%/95%/99% of the total variation (i.e. sum of all eigenvalues).

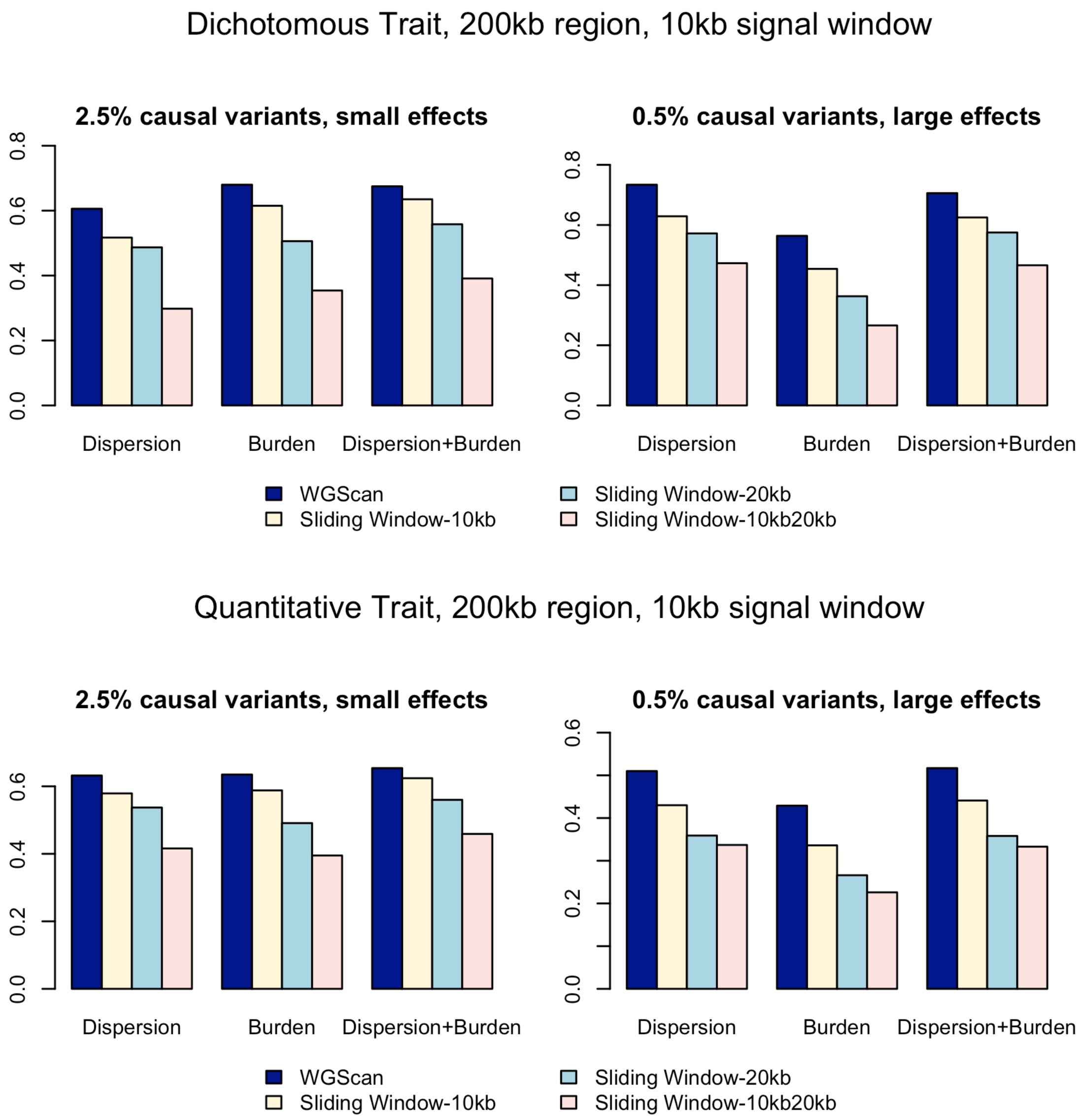

Supplementary Figure 2 Power simulation studies based on 1000 replicates. The region size is 200kb and there are 2.5% or 0.5% causal variants that reside in a 10kb signal window. WGScan: proposed test with candidate window sizes 5kb, 10kb, 15kb, 20kb, 25kb, 50kb; Dispersion-sliding, Burden-sliding or Dispersion+Burden-sliding: SKAT, Burden or SKAT-O test is applied to scan the region continuously using a sliding window of 10kb, 20kb or both, adjusted by Bonferroni correction for the total number of windows tested. For all methods we incorporate the Beta (1,25) weight to up-weight variants with lower minor allele frequency.

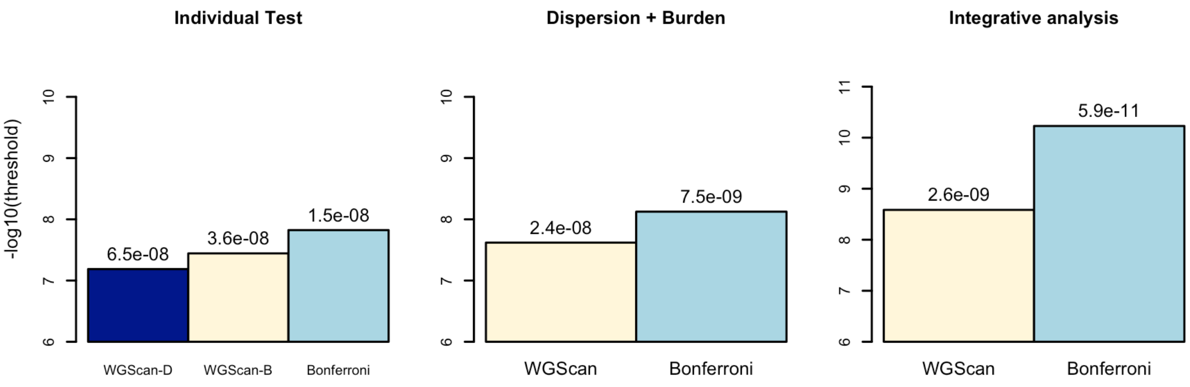

Supplementary Figure 3 Estimated significance thresholds for whole-genome sequencing studies (based on the Simons Simplex Collection data). Bars present estimated significance thresholds (-log10 scale) for different tests. WGScan-D/B: WGScan with dispersion/burden tests. The Bonferroni threshold is defined as 0.05 divided by the total number of tests. The significance threshold of dispersion + burden tests is for the minimum p-value of dispersion and burden tests (2 tests per window). In the right panel, 127 tissue-specific GenoNet scores are integrated, in addition to the original burden and dispersion tests (for a total of 256 tests per window).

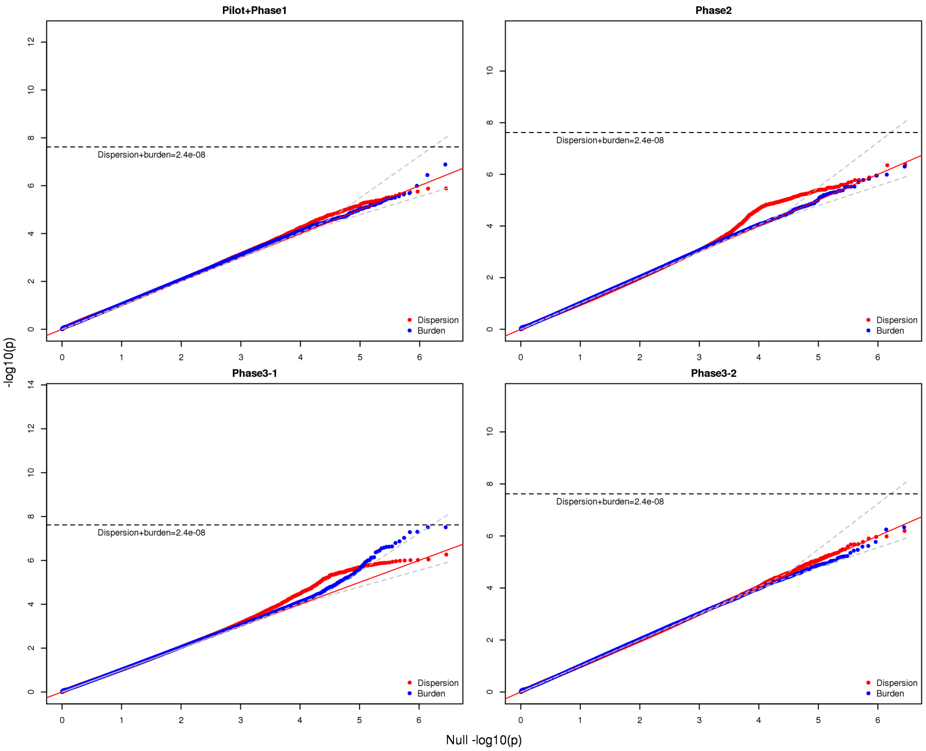

Supplementary Figure 4 Q-Q plot of the analysis of the whole-genome sequencing dataset from the Simons Simplex Collection. Each dot corresponds to a window. The dashed lines represent the WGScan threshold for the combination of dispersion and burden tests (2 tests per window).

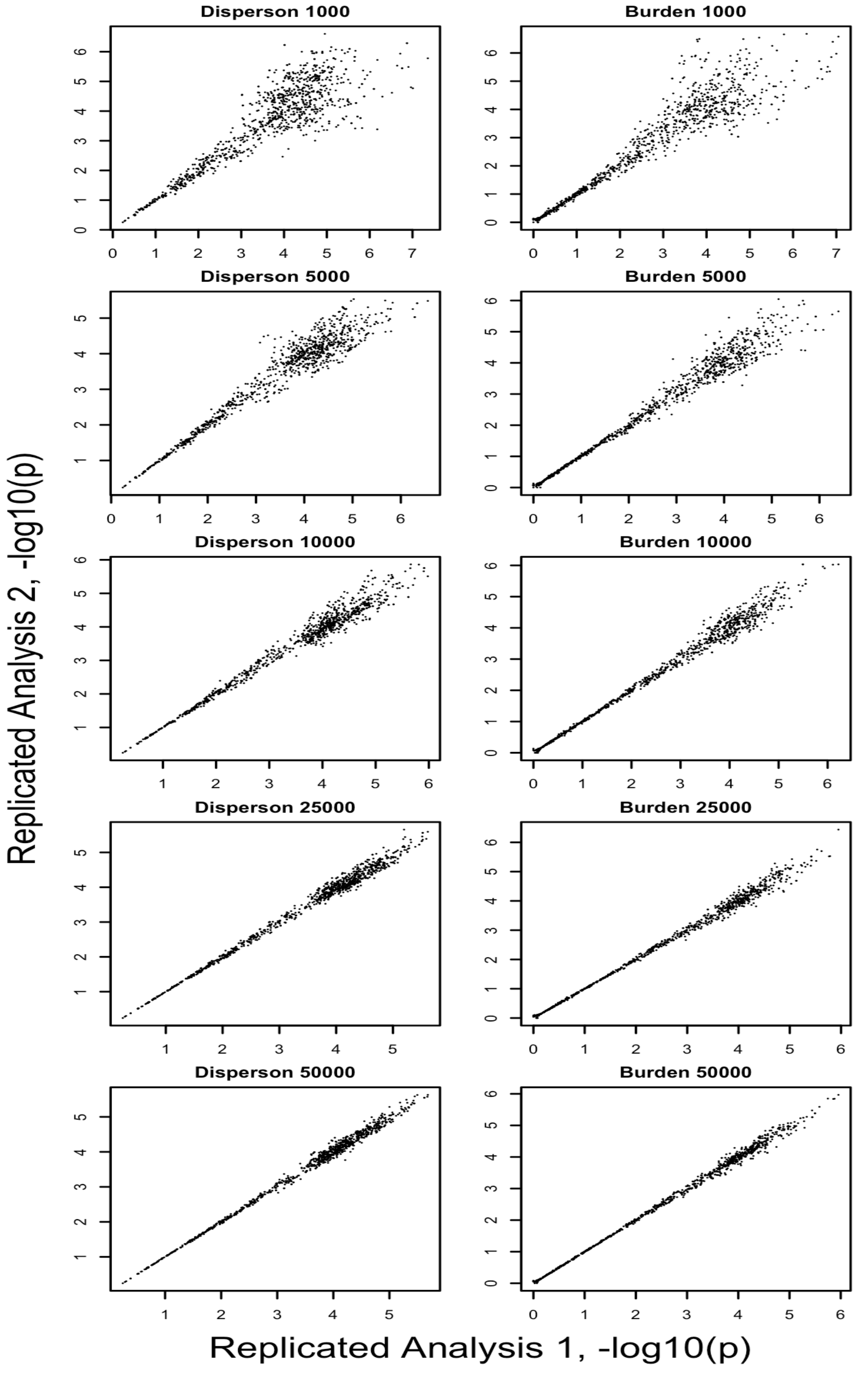

Supplementary Figure 5 Concordance between two replication analyses using the proposed moment-matching resampling approach to calculate window-based p-values. Each dot corresponds to a window that has either dispersion or burden p-value less than 0.0001 in the original genome wide scan.

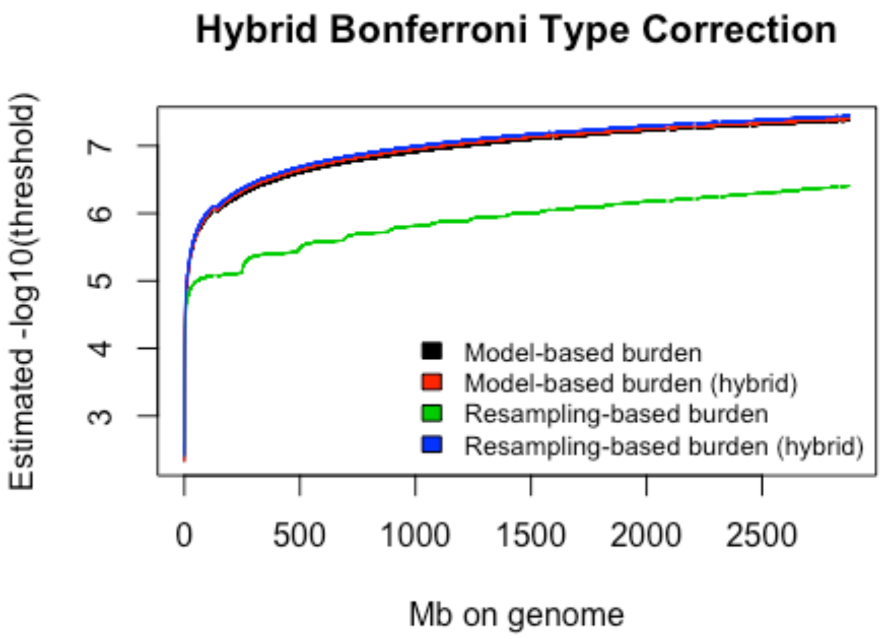

Supplementary Figure 6 Consistency between resampling-based hybrid Bonferroni type correction and unified model-based inference for the burden test. Model-based/resampling-based burden: the threshold is estimated based on the minimum p-value of model-based/resampling-based burden tests for the whole genome. Model-based/resampling-based burden (hybrid): the genome is first divided into 200kb regions. Then the threshold is estimated based on the minimum p-value of model-based/resampling-based burden tests for each region, and then the hybrid Bonferroni type correction is applied.

Supplementary Tables

Supplementary Table 1 Regions associated with lipid traits. For each trait, a significance threshold (1.04e-05) is estimated for the minimum p-value of original dispersion and burden tests scanning all regions, with candidate window sizes 5kb, 10kb, 15kb, 20kb, 25kb and 50kb, with half of the window overlapping with adjacent windows on each side. In addition, a significance threshold (3.75e-06) is estimated for the minimum p-value of original dispersion and burden tests and all tests weighted by GenoNet scores across 127 tissues (in total 256 tests per window).

Trait	Region	Index SNP	Window	Dispersion	Burden	MinP With GenoNet	Overlapping Gene	Overlapping Gene Range
hdl	16:56982818-57007610	rs3764261	56985640-57000221	5.2E-16	4.1E-04	5.2E-16	CETP	56995834-57017756
hdl	15:58670966-58696300	rs1532085	58681277-58691208	6.6E-05	3.0E-01	1.2E-07	ALDH1A2	58245622-58790065
ldl	1:109789346-109827253	rs599839	109810201-109815133	1.0E-05	1.6E-06	1.4E-06	CELSR2	109792640-109818378
ldl	19:45396898-45444742	rs4420638	45399344-45403924	5.6E-15	1.5E-01	5.7E-34	TOMM40	45394476-45406946
ldl	19:11182390-11212763	rs6511720	11192226-11207516	2.6E-07	9.2E-01	1.7E-07	LDLR	11200037-11244505
ldl	1:55497135-55513521	rs2479409	55500978-55505804	6.2E-25	2.4E-02	3.2E-30	PCSK9	55505148-55530526
chol	19:45396898-45444742	rs4420638	45399344-45403924	4.2E-06	4.5E-01	1.9E-13	TOMM40	45394476-45406946
chol	19:11182390-11212763	rs6511720	11192226-11207516	8.6E-06	4.9E-01	2.0E-06	LDLR	11200037-11244505
chol	1:55497135-55513521	rs2479409	55500978-55505804	5.3E-13	1.1E-01	7.2E-16	PCSK9	55505148-55530526
tg	11:116525729-116670738	rs964184	116611457-116624601	1.7E-10	4.7E-09	4.1E-21	BUD13	116618885-116643714
tg	8:19728585-19967629	rs12678919	19822741-19826992	4.2E-07	7.7E-01	1.5E-09	LPL	19796581-19824770
tg	2:27547719-27934731	rs1260326	27667184-27687036	4.8E-04	1.8E-06	1.8E-06	IFT172	27667239-27712678

Supplementary Table 2 Computational time for WGScan. Each cell presents the average time in seconds to analyze a 200kb region (~4000 variants) with candidate window sizes 5kb, 10kb, 15kb, 20kb, 25kb and 50kb, based on 1,000 replicates. For a genome-wide analysis, WGScan can be applied to small regions (i.e. 200kb regions) in parallel. The results are for quantitative traits but those for dichotomous traits remain similar.

Sample size	5,000		10,000		50,000		100,000	
	Time (secs)	Peak RAM (MB)	Time (secs)	Peak RAM (MB)	Time (secs)	Peak RAM (MB)	Time (secs)	Peak RAM (MB)
Preliminary Fit	3.57	501.01	6.57	1001.81	28.69	5008.44	75.55	10016.77
Dispersion	9.02	1166.52	13.14	1600.13	42.19	7049.85	106.95	14101.40
Burden	8.97	1166.92	13.07	1518.75	41.92	7045.31	105.97	14092.36
Dispersion+Burden	12.90	1256.28	17.14	1599.41	46.12	7045.29	112.34	14092.34

Supplemental Methods

Existing scan statistics

To allow for the simultaneous detection of the existence and location of associations, it has been suggested to use scan statistics. Scan statistics can be either competitive or self-contained. Competitive scan statistics aim to find the window within a larger region where risk variants cluster in a significant way compared with the rest of the region.[1,2,3] These tests are powerful if risk variants are enriched in a single window within the target region, but can lose power when there are multiple clusters of risk variants. Self-contained scan statistics have also been proposed in genetics, for example in DNA copy number analysis. Several authors have proposed to use the mean of marginal test statistics as a scan statistic.[4,5,6] These tests assume all variants in the set are associated with the outcome with the same direction; however, violation of this assumption leads to substantial loss of power. Li et al (2017) proposed a scaled quadratic scan statistic procedure (Q-SCAN) for genome-wide association studies (GWAS) of common variants, which is robust to positive and negative effects of genetic variants, and robust to the mixture of signal and null variants.[7] Although they derived a theoretical bound to asymptotically control the family wise error rate, the bound is not optimal and therefore the test can be conservative. In practice, they have to rely on an empirical threshold based on Monte Carlo simulations. In addition, Q-SCAN requires properly choosing a scale parameter prior to analysis to avoid inflation of scan statistics purely due to increasing the window size, but the optimal choice is often unknown.

Derivation of the significance threshold

As described in the Methods section, $T = \max_{k,l}[-\log(p_{\Phi_{kl}})]$ follows the Gumbel distribution with parameters v and ζ, and hence

$$P(T > x) = 1 - \exp\left[-\exp\left(-\frac{x - v}{\zeta}\right)\right].$$

Therefore the 0.95-quantile of $T = \max_{k,l}[-\log(p_{\Phi_{kl}})]$ equals

$$T_{0.95} = \hat{v} - \hat{\zeta}\log[-\log(0.95)].$$

Then the 0.05-quantile of $\min_{k,l} p_{\Phi_{kl}}$ equals

$$\alpha^* = \exp(-T_{0.95}) = \exp\{\hat{\zeta}\log[-\log(0.95)] - \hat{v}\}.$$

<u>Correlation between dispersion/burden statistics of adjacent windows</u>

We consider a simplified scenario $X_1, \dots, X_m, \dots, X_{2m}, \dots, X_{3m} \sim i.i.d.\, N(0,1)$, where $X_1, \dots, X_{3m}$, represent score statistics of $3m$ contiguous variants. We consider dispersion statistics $\sum_{1\le i\le 2m} X_i^2$ and $\sum_{m+1\le i\le 3m} X_i^2$, burden statistics $(\sum_{1\le i\le 2m} X_i)^2$ and $(\sum_{m+1\le i\le 3m} X_i)^2$, which correspond to windows with half the variants in a window overlapping with adjacent windows. Then we have

$$cor\left(\sum_{1\le i\le 2m} X_i^2, \sum_{m+1\le i\le 3m} X_i^2\right) = \frac{cov(\sum_{1\le i\le 2m} X_i^2, \sum_{m+1\le i\le 3m} X_i^2)}{var(\sum_{1\le i\le 2m} X_i^2)} = \frac{\sum_{m+1\le i\le 2m} var(X_i^2)}{\sum_{1\le i\le 2m} var(X_i^2)} = \frac{1}{2},$$

$$cor[(\sum_{1\le i\le 2m} X_i)^2, (\sum_{m+1\le i\le 3m} X_i)^2] = \frac{cov[(\sum_{1\le i\le 2m} X_i)^2, (\sum_{m+1\le i\le 3m} X_i)^2]}{var[(\sum_{1\le i\le 2m} X_i)^2]} =$$

$$\frac{cov(\sum_{1\le i,j\le 2m} X_i X_j, \sum_{m+1\le i,j\le 3m} X_i X_j)}{var(\sum_{1\le i,j\le 2m} X_i X_j)} = \frac{var(\sum_{m+1\le i,j\le 2m} X_i X_j)}{var(\sum_{1\le i,j\le 2m} X_i X_j)} =$$

$$\frac{var(\sum_{1\le i,j\le m} X_i X_j)}{var(\sum_{1\le i,j\le m} X_i X_j + \sum_{1\le i\le m, m+1\le j\le 2m} X_i X_j + \sum_{m+1\le i\le 2m, 1\le j\le m} X_i X_j + \sum_{m+1\le i,j\le 2m} X_i X_j)} =$$

$$\frac{var(\sum_{1\le i,j\le m} X_i X_j)}{2var(\sum_{1\le i,j\le m} X_i X_j) + 4var(\sum_{1\le i\le m, m+1\le j\le 2m} X_i X_j)} = \frac{var(X_1^2) + (m-1)var(X_1 X_2)}{2var(X_1^2) + 2(3m-1)var(X_1 X_2)}.$$

Since $var(X_1^2) = 2$, $var(X_1 X_2) = 1$,

$$cor[(\sum_{1\le i\le 2m} X_i)^2, (\sum_{m+1\le i\le 3m} X_i)^2] = \frac{m+1}{6m+2} < \frac{1}{2}.$$

The result shows that, under these simplified assumptions, correlations among p-values of dispersion statistics from adjacent windows tend to be larger than those of burden statistics, concordant with our empirical observations.

<u>Connection between score test with adjustment using parental genotypes and retrospective paired t-test for discordant sib-pair association</u>

For simplicity of notations, we assume that there is no covariate, i.e. $\hat{\mu}_i = \bar{Y} = \frac{1}{2}$. The score statistic for variant j can be written as

$$S_j = \sum_{i=1}^{n} \tilde{G}_{case,ij}\left(Y_{case,i} - \bar{Y}\right) + \sum_{i=1}^{n} \tilde{G}_{control,ij}\left(Y_{control,i} - \bar{Y}\right) = \frac{1}{2}\sum_{i=1}^{n} \tilde{G}_{case,ij} - \frac{1}{2}\sum_{i=1}^{n} \tilde{G}_{control,ij}$$

$$= \frac{n}{2}\left(\bar{\tilde{G}}_{case,j} - \bar{\tilde{G}}_{control,j}\right)$$

where $\tilde{G}_{case,ij} = G_{case,ij} - \frac{(P_{ij,F}+P_{ij,M})}{2}$ or similarly $\tilde{G}_{control,ij}$ is the adjusted genotype for the case or control in i-th family; $P_{ij,F}$ and $P_{ij,M}$ are the parental genotypes. The above equation shows that the score test is equivalent to a two-sample t-test that compares cases with controls. In addition, we can show that the adjusted genotype of a case is marginally uncorrelated with its sibling control

$$cov\left(\tilde{G}_{case,ij}, \tilde{G}_{control,ij}\right)$$

$$= E\left(cov\left(\tilde{G}_{case,ij}, \tilde{G}_{control,ij} | P_{ij,F}, P_{ij,M}\right)\right)$$

$$+ cov\left(E\left(\tilde{G}_{case,ij} | P_{ij,F}, P_{ij,M}\right), E\left(\tilde{G}_{control,ij} | P_{ij,F}, P_{ij,M}\right)\right) = 0,$$

because $\tilde{G}_{case,ij}$ and $\tilde{G}_{control,ij}$ are conditionally independent given parental genotypes, and $E\left(\tilde{G}_{case,ij} | P_{ij,F}, P_{ij,M}\right) = E\left(\tilde{G}_{control,ij} | P_{ij,F}, P_{ij,M}\right) = 0$. This further demonstrates that this two-sample t-test is equivalent to a paired t-test. Therefore, the score test with adjusted genotypes is

equivalent to a retrospective paired t-test that takes into account of the correlation between sib-pairs.

Supplemental References

1. Ionita-Laza, I., Makarov, V., Buxbaum, J.D. and ARRA Autism Sequencing Consortium (2012). Scan-statistic approach identifies clusters of rare disease variants in LRP2, a gene linked and associated with autism spectrum disorders, in three datasets. The American Journal of Human Genetics, 90(6), 1002-1013.
2. Ionita-Laza, I., Xu, B., Makarov, V., Buxbaum, J.D., Roos, J.L., Gogos, J.A., Karayiorgou M. (2014). Scan statistic-based analysis of exome sequencing data identifies FAN1 at 15q13.3 as a susceptibility gene for schizophrenia and autism. Proc Natl Acad Sci USA. 111: 343-348.
3. McCallum, K.J. and Ionita-Laza, I. (2015). Empirical Bayes scan statistics for detecting clusters of disease risk variants in genetic studies. Biometrics, 71(4), 1111-1120.
4. Arias-Castro, E., Donoho, D.L. and Huo, X. (2005). Near-optimal detection of geometric objects by fast multiscale methods. IEEE Transactions on Information Theory, 51(7), 2402-2425.
5. Zhang, N.R., Siegmund, D.O., Ji, H. and Li, J.Z. (2010). Detecting simultaneous changepoints in multiple sequences. Biometrika, 97(3), 631-645.
6. Jeng, X.J., Cai, T.T. and Li, H. (2012). Simultaneous discovery of rare and common segment variants. Biometrika, 100(1), 157-172.
7. Li, Z. and Lin, X. (2017). Simultaneous Detection of Signal Regions With Applications in Genome-Wide Association Studies. arXiv preprint arXiv:1710.05021.
8. Werling, D.M., Brand, H., An, J.Y., Stone, M.R., Zhu, L., Glessner, J.T., Collins, R.L., Dong, S., Layer, R.M., Markenscoff-Papadimitriou, E. and Farrell, A. (2018). An analytical framework for whole-genome sequence association studies and its implications for autism spectrum disorder. Nat Genet, 50, 727-736.
