# Supplementary material for: A genome-wide scan statistic framework for whole-genome sequence data analysis
Source: Nat Commun. 2019 Jul 9;10:3018. doi: 10.1038/s41467-019-11023-0 (PMC6616627; doi:10.1038/s41467-019-11023-0)
Supplement: Supplementary file 2 — Reporting Summary [file 41467_2019_11023_MOESM2_ESM.pdf]

nature research

Corresponding author(s): Iuliana Ionita-Laza

Last updated by author(s): May 20, 2019

Reporting Summary

Nature Research wishes to improve the reproducibility of the work that we publish. This form provides structure for consistency and transparency in reporting. For further information on Nature Research policies, see Authors & Referees and the Editorial Policy Checklist.

Statistics

For all statistical analyses, confirm that the following items are present in the figure legend, table legend, main text, or Methods section.

n/a	Confirmed	
☐	☒	The exact sample size (*n*) for each experimental group/condition, given as a discrete number and unit of measurement
☒	☐	A statement on whether measurements were taken from distinct samples or whether the same sample was measured repeatedly
☐	☒	The statistical test(s) used AND whether they are one- or two-sided<br>*Only common tests should be described solely by name; describe more complex techniques in the Methods section.*
☐	☒	A description of all covariates tested
☐	☒	A description of any assumptions or corrections, such as tests of normality and adjustment for multiple comparisons
☐	☒	A full description of the statistical parameters including central tendency (e.g. means) or other basic estimates (e.g. regression coefficient) AND variation (e.g. standard deviation) or associated estimates of uncertainty (e.g. confidence intervals)
☐	☒	For null hypothesis testing, the test statistic (e.g. *F*, *t*, *r*) with confidence intervals, effect sizes, degrees of freedom and *P* value noted<br>*Give P values as exact values whenever suitable.*
☒	☐	For Bayesian analysis, information on the choice of priors and Markov chain Monte Carlo settings
☒	☐	For hierarchical and complex designs, identification of the appropriate level for tests and full reporting of outcomes
☒	☐	Estimates of effect sizes (e.g. Cohen's *d*, Pearson's *r*), indicating how they were calculated

Our web collection on statistics for biologists contains articles on many of the points above.

Software and code

Policy information about availability of computer code

Data collection	The main focus of this paper is to propose a statistical method, with applications to existing datasets. Therefore no software was used to collect the data.
Data analysis	The data was analyzed by the proposed scan statistic method. The R package is available at http://www.funlda.com/toolkit, and has been submitted to CRAN.

For manuscripts utilizing custom algorithms or software that are central to the research but not yet described in published literature, software must be made available to editors/reviewers. We strongly encourage code deposition in a community repository (e.g. GitHub). See the Nature Research guidelines for submitting code & software for further information.

Data

Policy information about availability of data

All manuscripts must include a data availability statement. This statement should provide the following information, where applicable:

- Accession codes, unique identifiers, or web links for publicly available datasets
- A list of figures that have associated raw data
- A description of any restrictions on data availability

All data generated during this study are included in this published article (and its supplementary information files). Approved researchers can obtain the SSC population dataset described in this study (https://www.sfari.org/resource/simons-simplex-collection/) by applying at https://base.sfari.org. All other relevant data is available from the corresponding author on reasonable request.

Field-specific reporting

Please select the one below that is the best fit for your research. If you are not sure, read the appropriate sections before making your selection.

☒ Life sciences ☐ Behavioural & social sciences ☐ Ecological, evolutionary & environmental sciences

For a reference copy of the document with all sections, see nature.com/documents/nr-reporting-summary-flat.pdf

Life sciences study design

All studies must disclose on these points even when the disclosure is negative.

Sample size	The main focus of this paper is to propose a statistical method, with applications to existing datasets. Therefore no sample size calculation was performed. The Metabochip data for lipid traits was collected in Northern and Western Europe, for a total of 12,281 individuals. The whole-genome data from the Simon Simplex Collection consists of 7,144 individuals from 1,786 quad families.
Data exclusions	For the Metabochip data, we excluded samples and SNPs with call rates < 98% and used the complete data for each trait when there are missing outcomes. For the data from the Simon Simplex Collection, we focus on variant- and genotype-level quality metrics obtained from the VCF. We exclude variants with QUAL (quality score) < 200 or ReadPosRankSum < -1.4 (rank sum test for relative positioning of reference to alternative alleles within reads) or SOR (strand bias estimated by the symmetric odds ratio test) > 2.5 or GQ_MEAN (genotype mean quality) < 50.0 or QD (quality by depth) < 10.0 or AN (allele number) < 4152. For heterozygous genotypes, we exclude genotypes (these values will be treated as missing) with GQ (genotype quality of individual samples) < 99 or DP (coverage) < 10 or AB (allele balance) < 0.22 or AB > 0.78. For homozygous genotypes, we exclude genotypes with GQ (genotype quality of individual samples) < 30 or DP (coverage) < 18 or AB (allele balance) < 0.95. The thresholds were selected by Werling et al. (2018) through assessment of sequential receiver operating characteristic (ROC) curves generated from the true positive and true negative calls for each quality metric.
Replication	Not applicable.
Randomization	Not applicable.
Blinding	Not applicable

Reporting for specific materials, systems and methods

We require information from authors about some types of materials, experimental systems and methods used in many studies. Here, indicate whether each material, system or method listed is relevant to your study. If you are not sure if a list item applies to your research, read the appropriate section before selecting a response.

Materials & experimental systems

n/a	Involved in the study	
☒	☐	Antibodies
☒	☐	Eukaryotic cell lines
☒	☐	Palaeontology
☒	☐	Animals and other organisms
☒	☐	Human research participants
☒	☐	Clinical data

Methods

n/a	Involved in the study	
☒	☐	ChIP-seq
☒	☐	Flow cytometry
☒	☐	MRI-based neuroimaging
